# The Safety of Chemotherapy for Ovarian Malignancy during Pregnancy

**DOI:** 10.3390/jcm11247520

**Published:** 2022-12-19

**Authors:** Naidong Xing, Lihui Wang, Xinlei Sui, Chunru Zhao, Yan Huang, Jin Peng

**Affiliations:** 1 Department of Urology, Qilu Hospital of Shandong University, Jinan 250001, China; 2 Gynecology Center, Qingdao Women and Children’s Hospital, Qingdao 266034, China; 3 Center for Reproductive Medicine, Cheeloo College of Medicine, Shandong University, Jinan 250001, China; 4 Department of Obstetrics and Gynecology, Qilu Hospital of Shandong University, Jinan 250001, China

**Keywords:** ovarian malignancy, pregnancy, chemotherapy, safety

## Abstract

Background: Data on epidemiologic features, treatments and outcomes in women diagnosed with ovarian malignancy during pregnancy are very sparse due to its low incidence. The goal of our study was to summarize the epidemiologic characteristics of pregnant women complicated with ovarian malignancy and investigate the safety and efficacy of chemotherapy during pregnancy. Methods: We retrospectively analyzed the clinicopathological data of eight patients suffering from ovarian malignancy during pregnancy in our institution from June 2011 to July 2021. Furthermore, a systematic literature search was conducted in PubMed up to 1 September 2021, which identified 92 cases with ovarian malignancy during pregnancy eligible for the analysis. Therefore, we collected the data of 100 pregnant patients complicated with ovarian malignancy, including clinical demographics, tumor characteristics, treatment interventions and outcomes. Results: In total, 100 pregnant patients complicated with ovarian malignancy were investigated and classified into three groups: 34 cases in the epithelial ovarian cancer (EOC) group, 38 cases in the germ cell tumors (GCTs) group and 28 cases in the sex cord-stromal tumors (SCSTs) group. The onset age of pregnant patients with epithelial ovarian cancer was significantly higher than that of other patients. Pelvic mass and abdominal pain were the common clinical presentations of pregnant patients with ovarian malignancy. For distinguishing epithelial ovarian cancer during pregnancy, the area under the curve (AUC) of CA-125 was 0.718 with an optimal cutoff value of 58.2 U/mL. Moreover, 53 patients underwent surgery during pregnancy, the majority of whom underwent unilateral adnexectomy in the second trimester. Furthermore, 43 patients received chemotherapy during pregnancy, and 28 delivered completely healthy newborns at birth; 13 neonates showed transient abnormalities without further complications; and 2 died during the neonatal period. Conclusions: Our study reveals the safety of chemotherapy for ovarian malignancy during pregnancy. However, large-sample prospective studies are still needed to further explore the safety of chemotherapy in pregnant patients with malignancy to choose the appropriate chemotherapy regimen and achieve the maximum benefit for patients.

## 1. Introduction

The prevalence of ovarian malignancy as a complication with pregnancy is continuously increasing due to increased maternal ages and the improvement of medical diagnostic technology [1]. The disease poses a major threat to maternal and fetal health. According to a population-based study, mortality was 4.7% in patients with ovarian cancer during pregnancy [2]. The majority of patients are asymptomatic, which may delay the diagnosis of ovarian malignancy. Therefore, the epidemiological characteristics of ovarian malignancy during pregnancy need to be investigated. When a malignancy during pregnancy is suspected, specific serum tumor markers can serve as tools in the initial diagnosis of patients with ovarian cancer. Cancer antigen 125 (CA-125) is proposed as a specific marker for epithelial ovarian cancer. Alpha-fetoprotein (AFP) has been shown to be expressed by germ cell tumors, specifically endodermal sinus tumors. However, due to multiple physiological changes that occur in pregnancy, such as the proliferation of embryonic tissue, maternal serum tumor marker levels can increase physiologically and fluctuate during pregnancy [3,4]. Furthermore, the levels of these tumor markers are also elevated when placental or fetal abnormalities occur (such as Down syndrome, open neural tube defects, preeclampsia, growth restriction and preterm labor) [4,5]. Thus, the reference ranges of nonpregnant patients are not fit for the diagnosis of patients with ovarian malignancy during pregnancy. At present, limited research is available on serum tumor markers during pregnancy, and there is no uniform cutoff value of AFP and CA-125 to diagnose ovarian malignancy in pregnancy. Thus, an adjusted cutoff level should be established to diagnose ovarian malignancy during pregnancy.

If CA-125 or AFP detects possible ovarian malignancy, an ultrasound may be helpful but is limited due to the occlusion of the growing fetus. If necessary, computerized tomography (CT) or magnetic resonance imaging (MRI) could even be performed for patients during late pregnancy. Once the diagnosis is established, the management of ovarian malignancy in pregnancy is complex and challenging, and this affects not only the prognosis of the mother but also the development of the fetus. Surgical removal remains the cornerstone in the treatment of ovarian malignancy during pregnancy with adequate preparation and careful monitoring. Surgery is usually performed in the second trimester, during which time the risk of spontaneous abortion can be reduced [6]. Whether surgery or chemotherapy should be performed first depends on the progression of the malignancy and the gestational age. Moreover, chemotherapy is also an important strategy for the management of ovarian malignancy patients during pregnancy. During pregnancy, the use of chemotherapy can increase the chances of fetal preservation [7]. However, due to their relatively small molecular weight, most chemotherapy drugs are capable of crossing the placenta and may induce fetal toxicity [6,8,9,10]. The scarce evidence on the fetal safety of maternal chemotherapy during pregnancy is limited to small retrospective studies and case reports, making their results difficult to interpret [9]. It is currently recognized that exposure to chemotherapy during the first trimester can increase the risk of major congenital malformations up to 10–20% [6,9,10,11]. Moreover, the efficacy and safety of chemotherapy during the second and third trimesters are still controversial.

In this study, we first identified the epidemiologic characteristics of pregnant women complicated with ovarian malignancy. We reveal an adjusted cutoff level tumor marker to help diagnose ovarian malignancy during pregnancy. Moreover, the safety of chemotherapy to the fetus during pregnancy was investigated.

## 2. Materials and Methods

**Figure 1:**
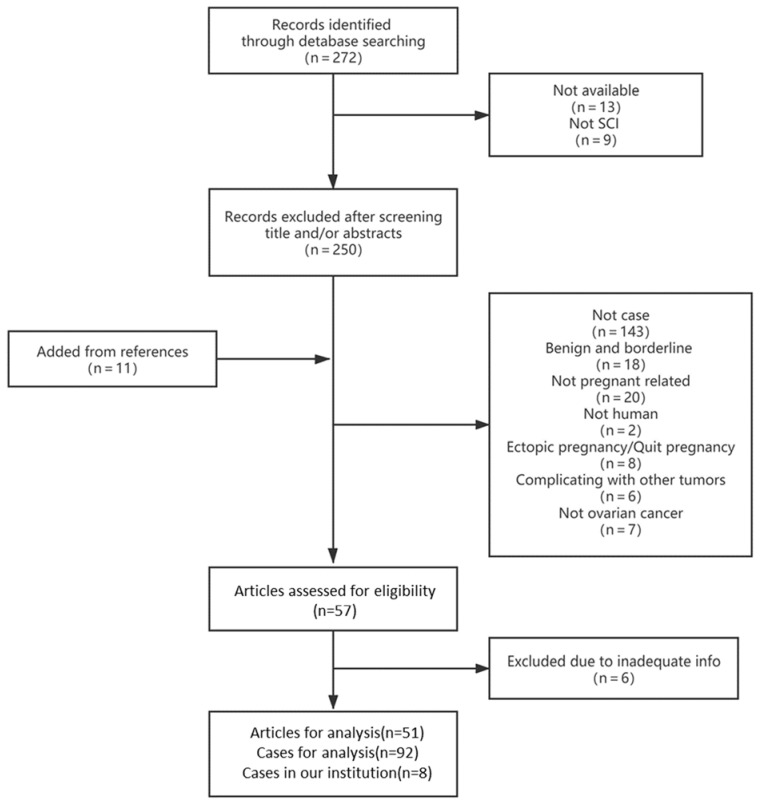
Flow chart showing the selection of the study population.

We retrospectively collected the medical records of patients diagnosed with malignant ovarian tumors during pregnancy in our institution from June 2011 to July 2021. Additionally, a detailed analysis of the literature was found in the PubMed databases using the keywords “ovarian cancer”, “ovarian malignancy”, “adnexal masses”, “ovarian tumor”, “germ cell tumor”, “endodermal sinus tumor”, “yolk sac tumor”, “sex cord-stromal tumor”, “granulosa cell tumor” and “pregnancy” or “pregnant”. The search was limited to articles published in English up to 1 September 2021. Inclusion criteria were defined as case reports or case series describing pregnant patients with ovarian malignancy, including descriptions of clinical demographics, tumor characteristics and treatment interventions. References for each evaluated article were also reviewed, and the corresponding articles that met the inclusion criteria were included in the study. Excluded articles included reviews and case reports in which there were inadequate clinical data. The screening process is shown in Figure 1. A total of 272 articles were searched from the databases, and 57 articles met our criteria. These articles were further assessed, and 6 articles were removed because of inadequate description of the cases. After reviewing the references from the eligible articles, 11 articles were added. Finally, 100 cases comprising 92 cases from 51 articles [12,13,14,15,16,17,18,19,20,21,22,23,24,25,26,27,28,29,30,31,32,33,34,35,36,37,38,39,40,41,42,43,44,45,46,47,48,49,50,51,52,53,54,55,56,57,58,59,60,61,62] and 8 cases in our institution were eligible for the study analysis.

Information regarding the clinical demographics including the areas of cases, maternal age at diagnosis, parity, gestational age at diagnosis and clinical presentation of patients was collected. Tumor characteristics included tumor markers, ascites, tumor laterality, histology type and stage. Pregnancy information included mode and timing of delivery, neonatal sex, infant weight, Apgar scores and serious adverse events. Information on surgical interventions and chemotherapy during pregnancy was investigated.

In our study, pregnancy trimesters were defined as first trimester—up to 12 weeks, second trimester—13–28 weeks and third trimester—after 28 weeks, respectively. We defined preterm-born and term-born birth at <37 and ≥37 weeks gestation, respectively. We considered stages I and II as early stages and stages III and IV as advanced stages. Tumor stage was determined according to International Federation of Gynecology and Obstetrics (FIGO) criteria version 2014 [63]. We defined puerperium as 6 weeks postpartum. Fertility-sparing surgery (FSS) after delivery was defined as surgery in which the uterus and at least one side of the ovary were preserved, while radical surgery was defined as surgery including hysterectomy with or without a complete staging operation. Maternal and fetal complications included tumor rupture or torsion, oligohydramnios, premature rupture of membrane (PROM), recurrence during pregnancy and neonatal abnormal development, such as malformation, respiratory distress, intrauterine growth restriction (IUGR), abnormal laboratory data, admission in neonatal intensive care unit and death. All patients had a pathologic diagnosis after surgical resection. All procedures were in accordance with the ethical standards of the national research committee and with the 1964 Helsinki Declaration and its later amendments.

### Statistical Analysis

For the data evaluation, descriptive analysis of characteristics of ovarian cancer during pregnancy was performed expressed with number (%) for categorical variables. The normality of continuous variables was tested by the Shapiro–Wilk test and described as the mean (SD) or median (range) as appropriate, and statistical significance was examined with Student’s t-test or Wilcoxon–Mann–Whitney test as appropriate. ANOVA was used to test maternal age of diagnosis among the three groups. The chi-square test was used to examine categorical variables. Receiver operator curve (ROC) curves were plotted, and the area under the curve (AUC) and 95% confidence interval (CI) were calculated for CA-125 and AFP. Sensitivity and specificity were used to describe predictive properties. *p* values of less than 0.05 were considered statistically significant (two-tailed). All statistical analyses were performed using Statistical Package for Social Scientists software (IBM SPSS 25.0, Armonk, NY, USA, SPSS Corp.).

## 3. Results

### 3.1. Epidemiological Characteristics of Pregnant Patients Complicated with Ovarian Malignancy

Among 100 patients complicated with ovarian malignancy enrolled in our study, 34 patients were included in the epithelial ovarian cancer (EOC) group, 38 patients were included in the germ cell tumors (GCTs) group and 28 patients were included in the sex cord-stromal tumors (SCSTs) group (Table 1). The majority of the cases came from Asia (41%) and North America (40%), while other cases came from Europe (18%) and Australia (1%). The mean maternal age at diagnosis was 32.8 years old for women with EOC, 26.9 for women with GCTs and 23.6 for women with SCSTs (Table 1). The onset age of pregnant patients with epithelial ovarian cancer was significantly higher than that of patients with other types of ovarian malignancies (*p* < 0.001, Table 1). 37% of the women were nulliparous, while 34% of the women were multiparous. In all cases, the most common clinical presentation was pelvic mass (36.4%), most of which were usually asymptomatic and detected incidentally by ultrasound (*p* = 0.036, Table 1). Abdominal pain was noted in 23.6% of patients, while abdominal distention was noted in 5.5% of patients (Table 1). Other symptoms included vaginal bleeding and virilization (Table 1). Unilateral tumors comprised 82% cases (Table 1). Meanwhile, 19% of cases showed ascites (Table 1). The diagnosis of pregnant patients complicated with ovarian malignancy was mostly in the second trimester of gestation, while other malignancies were accidentally found during cesarean section or puerperium (Table 1). Moreover, 66% of ovarian malignancies associated with pregnancy were diagnosed in the early stages (stage I or II), while 23 women were found to have an advanced stage (stage III or IV) (Table 1). However, nearly half of the patients with EOC during pregnancy were in an advanced stage (Table 1).

### 3.2. Tumor Markers Used to Diagnose Ovarian Malignancy in Patients during Pregnancy

**Figure 2:**
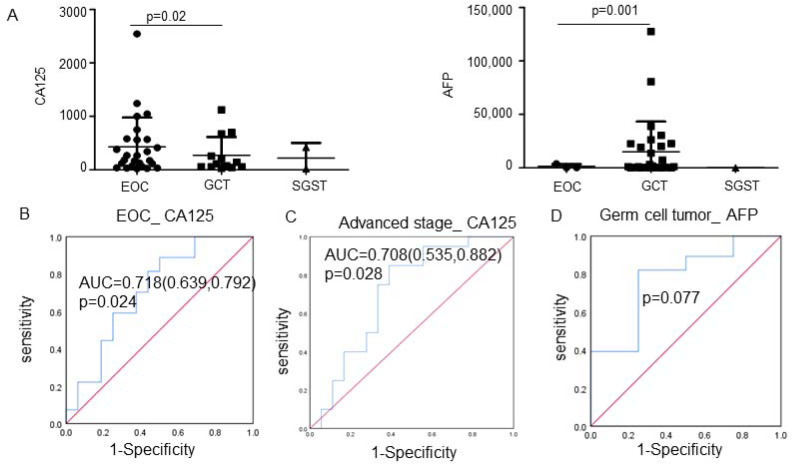
(**A**) The serum CA-125 and AFP levels in the EOC group, GCT group, SGST group and healthy control group. (**B**) Receiver operating characteristic curve of serum CA-125 in the diagnosis of patients with epithelial ovarian cancer. (**C**) Receiver operating characteristic curve of serum CA-125 to predict advanced-stage ovarian cancer during pregnancy. (**D**) Receiver operating characteristic curve of serum AFP in the diagnosis of patients with germ cell tumors during pregnancy. (bule line: ROC curve; red line: reference line) Abbreviations: EOC, epithelial ovarian cancer; GCTs, germ cell tumors; SCSTs, sex cord-stromal tumors; CA-125, cancer antigen 125; AFP, alpha-fetoprotein; AUC, area under the curve.

To facilitate early diagnosis, we explored tumor marker levels in pregnant patients with ovarian malignancy. The alpha-fetoprotein (AFP) and cancer antigen 125 (CA-125) levels of pregnant patients complicated with ovarian malignancy were estimated. The average concentrations of CA-125 in the EOC group were significantly higher than in the GCTs group (*p* = 0.02; Figure 2A). The AFP level of the GCTs group was also significantly higher than that of the EOC group (*p* = 0.001; Figure 2A). The optimal cutoff value of CA-125 for the prediction of pregnant females with epithelial ovarian cancer was 58.2 U/mL, with a sensitivity of 80.8% and specificity of 81.4% (*p* = 0.024, Figure 2B). Moreover, elevated CA-125 levels predicted an advanced stage in pregnant patients with EOC (AUC 0.708, 95% CI, 0.535–0.882, *p* = 0.028; Figure 2C). However, the serum AFP level could not distinguish pregnant patients with germ cell tumors (*p* = 0.077; Figure 2D).

### 3.3. Maternal and Neonatal Outcomes of Pregnant Patients Diagnosed with Ovarian Malignancy

Seventy percent of patients gave birth via cesarean section, and only 17 patients gave birth vaginally, while pregnant patients with EOC preferred to give birth by cesarean section (Table 2). There were 101 newborns due to one set of twins, while 40 neonates were preterm and 26 were full-term (Table 2). No significant differences were observed in birth weight, birth sex or Apgar score between the EOC group and GCT group (*p* = 0.127; 0.109; 0.276). The most common maternal complication was tumor rupture or torsion during pregnancy (*p* = 0.036, Table 2). Maternal death occurred in only one case due to endodermal sinus tumor recurrence and multiple metastases before chemotherapy treatment [12]. In terms of fetal complications, the most common transient abnormality was respiratory distress (6.93%), followed by routine blood abnormalities (3.96%) and intrauterine growth restriction (3.96%) (*p* = 0.042, Table 2). Fetal malformations occurred in three cases (Table 2) [25,44,57]. One fetus was exposed to the BEP regimen and then suffered from mild hypospadias with normal physical and neurological development [44]. One newborn who was exposed to carboplatin and paclitaxel had bilateral congenital talipes equinovarus with no further complications, and his maternal family history of congenital talipes equinovarus might be a significant factor [57]. One week after the BEP regimen in utero, one fetus was noted to have ventriculomegaly and cerebral atrophy via obstetrical ultrasound [25]. Two neonatal deaths were described: one newborn died 5 days after delivery due to multiple congenital anomalies that were diagnosed before starting chemotherapy [46], and another premature newborn died within the first week of life without definitive reasons [60]. In addition, one baby suffered from intussusception at 7.5 months of age (Table 2) [33].

### 3.4. The Management of Pregnant Patients with Ovarian Malignancy

To figure out the factors related to the outcome of fetal births, we further estimated the treatment strategy of pregnant patients with ovarian malignancy. Fifty-three patients received tumor reductive surgery during pregnancy, 77.4% of whom underwent surgery during the second trimester (Table 3). Unilateral adnexectomy was more commonly performed during gestation (Table 3). There were 43 cases exposed to chemotherapeutic drugs during pregnancy. The median gestational age was 22 weeks (14th–31st week) when chemotherapy was started (Table 3). Twenty-nine patients received chemotherapy agents in the second trimester, and three patients started chemotherapy in the third trimester (Table 3).

**Figure 3:**
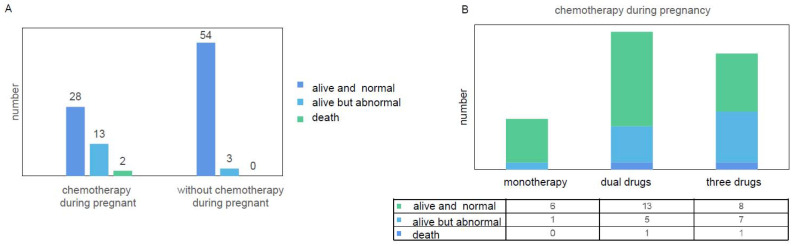
(**A**) Fetal outcomes in patients during pregnancy exposed to chemotherapy or not exposed to chemotherapy. (**B**) Fetal outcomes in pregnant patients with ovarian malignancy receiving different chemotherapy regimens.

Twenty-eight neonates who were exposed to chemotherapeutic agents in utero were discharged alive and without any neonatal complications (Figure 3A). However, 15 abnormal newborns were exposed to in utero chemotherapy. Among the 15 cases, 10 neonates showed transient abnormalities, including respiratory distress, routine blood abnormalities and neonatal infection, 3 had mild abnormal malformations without further complications, and 2 died during the neonatal period (Figure 3A).

To determine the effect of in utero chemotherapy on the fetus, we evaluated the chemotherapeutic regimens. Chemotherapeutic regimens included single-agent and double-agent combinations and three-drug combinations. The single-agent chemotherapeutic regimens included paclitaxel, carboplatin or cisplatin monotherapy. All the dual-drug regimens involved platinum-based chemotherapy except in the case of one patient who received Adriamycin and vincristine [49]. Three-drug combination regimens consisted of the BEP regimen (bleomycin/etoposide/cisplatin), PVB regimen (cisplatin/vincristine/bleomycin) or VAC regimen (vincristine/actinomycin/cyclophosphamide). Double-agent chemotherapeutic regimens were the most commonly (44.2%) used in pregnant patients, followed by three-drug chemotherapeutic regimens (37.2%) (Table 3). Among 16 patients who received three-drug combination regimens, 3 patients were treated with the PVB regimen, 3 patients were treated with the VAC regimen, and 10 patients received the BEP regimen during pregnancy.

Due to the detailed chemotherapy regimen of one case not being identified, 42 cases were included for analysis (Figure 3B). Twenty-seven pregnant patients who received chemotherapy delivered alive and normal fetuses (Figure 3B). Among 13 abnormal newborns, 1 was exposed to single-agent chemotherapy, 5 were exposed to dual-drug regimens and 7 were exposed to three-drug combinations. Among five newborns exposed to dual-drug regimens, one suffered malformation, and four newborns suffered transient abnormalities without further complications. The male neonate who was exposed to a dual-drug regimen (carboplatin and paclitaxel) had bilateral congenital talipes equinovarus, and his maternal family history of congenital talipes equinovarus might be a significant factor [57]. In the cases of seven abnormal newborns who were exposed to three-drug combinations, two newborns suffered malformations and five newborns suffered mild abnormalities without further complications. One fetus was exposed to the BEP regimen after the 21st week of gestation and suffered from mild hypospadias with normal physical and neurological development [44]. Another fetus exposed to the BEP regimen between 25 and 28 weeks gestational age developed ventriculomegaly in utero and subsequently cerebral atrophy [25]. There were two neonatal deaths: one newborn who was exposed to cisplatin and docetaxel died 5 days after delivery due to multiple congenital anomalies that were diagnosed before starting chemotherapy, and another premature newborn exposed to the BEP regimen died within the first week of life without definitive reasons (Figure 3B).

Moreover, forty-nine patients underwent additional secondary surgery after pregnancy. A total of 69.4% of patients underwent radical surgery, including hysterectomy with or without lymphadenectomy (Table 3). No radiotherapy was implemented during gestation, and only one patient received postpartum radiotherapy [51].

## 4. Discussion

The incidence of ovarian malignancy during gestation is low, and the majority of data came from case reports and lacked systematic epidemiological reviews. Here, we first identified the systematic epidemiological characteristics of pregnant patients with ovarian malignancy. We first reveal that Asian and North American patients had a higher incidence of ovarian malignancy during pregnancy, which may be due to a bias of the literature selection and low reported rates in certain countries. The question of whether ovarian stimulation caused by high hormone levels during pregnancy is a precipitating factor to ovarian malignancy during pregnancy needs further study. Unsurprisingly, the onset age of pregnant patients with epithelial ovarian cancer is significantly higher than that of other patients. The major clinical presentations of ovarian malignancy during pregnancy were pelvic mass and abdominal pain, similar to ovarian malignancy alone.

Due to the overlap between symptoms and physiological pregnancy changes, diagnosis is difficult. Therefore, we demonstrated here that the elevated CA-125 cutoff value of 58.2 U/mL was employed to predict ovarian malignancy in monitoring pregnant patients. The cutoff value in our study is similar to a previous report whose recommended cutoff value was ≥60 U/mL [4]. A previous study reported that maternal CA-125 values were highest in the first trimester, but elevated maternal CA-125 levels may also occur in the second and third trimesters of pregnancy [64]. However, another study showed that serum CA-125 levels were significantly elevated in the third trimester during pregnancy compared to the second trimester, while there was no significant difference between the first and second trimesters [3]. Therefore, the diagnostic utility of CA-125 in monitoring pregnant women with ovarian tumors must be considered carefully. Here, we demonstrate that optimal cutoff points of maternal serum CA-125 are useful in screening for epithelial ovarian cancer as a complication of pregnancy. According to our data, AFP is not a relatively reliable biomarker of pregnant patients with germ cell tumors. Moreover, maternal serum AFP has been widely used for identifying fetuses with open neural tube defects and chromosomal abnormalities. Furthermore, adverse pregnancy outcomes such as preeclampsia, premature birth, placental abruption and fetal demise have been shown to be associated with unexplainably high AFP in pregnancies [65,66,67].

The management of ovarian malignancy during pregnancy can be challenging because of the risk of fetal wastage and the possibility of surgery-related complications. The guidelines on gynecologic cancers in pregnancy demonstrate that surgery performed in the second trimester is preferable because during this period, the risk of miscarriage is decreased, and the size of the uterus still allows a certain degree of access [6]. Consistent with the guidelines, our data show that the majority of surgical cases were performed during the second trimester (77.4%). Furthermore, the preterm birth rate of pregnancies (39.6%) associated with malignancy is far greater than that in the general population (approximately 5%) [68], perhaps due to balancing maternal and infant benefits.

The safety of chemotherapy administered during pregnancy is still controversial [69]. The timing of chemotherapeutic exposure during pregnancy is critical. According to our data, no patient received chemotherapy in the first trimester. It is currently recognized that chemotherapeutic drug exposure during the first trimester may increase the risk of fetal malformations [9]. Therefore, chemotherapy should be avoided during the first trimester. Chemotherapy is recommended after 14 weeks of pregnancy but no more than 35 weeks of pregnancy [6]. There should be at least a 3-week window between the last cycle of chemotherapy and delivery, which is important to prevent myelosuppression in the mother and neonate [6,10]. Additionally, based on the guidelines of the third international consensus meeting of the European Society of Gynecological Oncology (ESGO), carboplatin and paclitaxel are recommended for use in epithelial ovarian cancer during pregnancy [6]. In germ cell tumors and sex stromal cell tumors, chemotherapy consists of the administration of paclitaxel and carboplatin or PVB or BEP regimens [10]. Several studies have reported that combination treatment with paclitaxel and carboplatin during pregnancy resulted in safety and good fetal outcomes [35,43,56,70,71].

A review of 376 fetuses exposed to chemotherapy in utero reported complications of 2.9% malformations, 5% fetal death, 1% neonatal death, 7% IUGR, 5% preterm birth and 4% transient myelosuppression [72]. We have demonstrated here that there was 1% neonatal mortality after in utero chemotherapy, which was lower than 17‰ among the general population globally [73]. According to our data, it seems that more chemotherapeutic drugs during pregnancy are associated with a higher risk of fetal abnormalities. In terms of three fetal malformation cases, one newborn who had bilateral congenital talipes equinovarus was exposed to carboplatin and paclitaxel during the second trimester. Congenital talipes equinovarus is a common birth defect with a prevalence of 1 to 2 per 1000 live births, and the etiology of this condition remains unclear [74]. Two newborns exposed to the BEP regimen in the uterus had malformations. The newborn suffering from mild hypospadias was exposed to the BEP regimen after 21 weeks of gestation [44]. Since urethral development begins in utero at approximately 8 weeks and is completed by 15 weeks of gestation [44], we would suggest that the hypospadias in this case was not correlated with chemotherapy. Another fetus was exposed in utero to one cycle of the BEP regimen during the second trimester and developed ventriculomegaly secondary to cerebral atrophy [25]. However, there remains no clear reason for the occurrence of ventriculomegaly, and there are controversies. A previous study showed that the total dose of etoposide per unit time (100 mg/m^2^ daily for 5 days) used in the patient impacted fetal brain development [25]. In contrast, it has also been reported that cerebral atrophy is unlikely secondary to in utero exposure to chemotherapy with BEP [33]. In the cases of the two neonatal deaths, one newborn died 5 days after delivery due to multiple congenital anomalies that were diagnosed before starting chemotherapy. The other newborn exposed to BEP chemotherapy died within one week without clear reasons. Therefore, whether the occurrence of newborn death was associated with chemotherapy remains unknown.

In accordance with previous studies, exposure to chemotherapy in the second and third trimesters of pregnancy does not increase the risk of fetal malformations and newborn deaths [7,8,75,76]. Studies have revealed that chemotherapy has no clear adverse effects on postnatal growth or cognitive or cardiac function [8,75]. Antenatal chemotherapy exposure was associated with an increased risk of lower gestational age and neonatal intensive care admission [76,77]. Therefore, it is important to offer optimal chemotherapy at an appropriate time to the mother without placing the fetus at serious risk.

## 5. Conclusions

Higher cutoff values of CA-125 could be used as tumor markers in pregnant patients with epithelial ovarian cancer. Chemotherapy may be administered during the second and third trimesters without an apparently higher risk for fetal complications. Antenatal chemotherapy exposure requires a balance between risks and benefits for the mother and fetus.

## Figures and Tables

**Table 1:** Epidemiological characteristics of pregnant patients complicated with ovarian malignancy.

	Epithelial Ovarian Cancer Group (n = 34)	Germ Cell Tumors Group (n = 38)	Sex Cord-Stromal Tumors Group (n = 28)	
Area of cases				*p* = 0.035
Asia	15	23	3	41(41%)
Europe	11	7	0	18(18%)
North America	7	8	25	40(40%)
Australia	1	0	0	1(1%)
Maternal age at diagnosis	32.8 ± 5.6	26.9 ± 4.7	23.6 ± 4.3	*p* < 0.001
<20	0	2	4	6(6%)
20–29	8	24	22	54(54%)
≥30	26	12	2	40(40%)
Multipara				*p* = 0.135
Yes	11	8	15	34(34%)
No	16	13	8	37(37%)
Missing	7	17	5	29(29%)
Clinical presentation				*p* = 0.036
Abdominal pain	6	11	9	26(23.6%)
Abdominal distention	1	4	1	6(5.5%)
Pelvic mass	22	13	5	40(36.4%)
Vaginal bleeding	1	1	1	3(2.7%)
Virilization	0	0	3	3(2.7%)
Missing	6	14	12	32(29.1%)
Ascites				*p* = 0.234
Yes	8	9	2	19(19%)
No	4	3	24	31(31%)
Missing	22	26	2	50(50%)
Gestational age at diagnosis				*p* = 0.027
Before pregnancy ^a^	0	3	0	3(3%)
1st trimester	5	0	2	7(7%)
2nd trimester	16	24	1	41(41%)
3rd trimester	0	1	0	1(1%)
Postpartum ^b^	11	8	15	34(34%)
Missing	2	2	10	14(14%)
Tumor laterality				*p* = 0.033
Unilateral	23	32	27	82(82%)
Bilateral	4	2	1	7(7%)
Missing	7	4	0	11(11%)
Tumor stage				*p* = 0.027
I-II	18	23	25	66(66%)
III-IV	14	8	1	23(23%)
Missing	2	7	2	11(11%)

Notes: Numbers with percentages or mean with SD are shown; ^a^ Diagnosed before pregnancy and recurrence during pregnancy; ^b^ Diagnosed disease during cesarean section or puerperium.

**Table 2:** Maternal and neonatal outcomes of pregnant patients complicated with ovarian malignancy.

	Epithelial Ovarian Cancer Group (n = 34)	Germ Cell Tumors Group (n = 38)	Sex Cord-Stromal Tumors Group (n = 28)	
Mode of delivery				*p* = 0.021
Vaginal delivery	2(6.0%)	13(34.2%)	2(7.2%)	17(17%)
Cesarean section	32(94.0%)	25(65.8%)	13(46.4%)	70(70%)
Missing	0	0	13(46.4%)	13(13%)
Timing of birth				*p* = 0.032
Preterm	17	21	2	40(39.6%)
Full-term	11	13	2	26(25.7%)
Missing	6	5	24	35(34.7%)
Birth weight (g)	2595.7 ± 656.7	2341.8 ± 614.2	-	*p* = 0.127
Sex of neonate				*p* = 0.109
Male	16	12	1	29(28.7%)
Female	10	11	2	23(22.8%)
Missing	8	16	25	49(48.5%)
Apgar scores				*p* = 0.276
1 min	9	9	-	
5 min	9	10	-	
Maternal complications				*p* = 0.036
Rupture or torsion of tumor	10	9	7	26(26%)
Oligohydramnios	1	4	0	5(5%)
Premature rupture of membrane	3	1	0	4(4%)
Recurrence during pregnancy	1	4	1	6(6%)
Maternal death	0	1	0	1(1%)
Fetal complications				*p* = 0.042
Fetal malformations	1	2	0	3(2.97%)
Neonatal death	1	1	0	2(1.98%)
Respiratory distress	1	6	0	7(6.93%)
Routine blood abnormality	0	4	0	4(3.96%)
Neonatal infection	1	0	0	1(0.99%)
Neonatal intensive care unit	1	0	0	1(0.99%)
Intrauterine growth restriction	0	4	0	4(3.96%)
Intussusception	0	1	0	1(0.99%)

Notes: Numbers with percentages or mean with SD are shown.

**Table 3:** Management of pregnant patients complicated with ovarian malignancy.

	N (%)
Gestational age at surgery during pregnancy	53
1st	7(13.2%)
2nd	41(77.4%)
3rd	1(1.9%)
Missing	4(7.5%)
Adnexal surgery during pregnancy	53
Cystectomy	2(3.8%)
Unilateral	41(77.4%)
Bilateral	6(11.2%)
Missing	4(7.6%)
Chemotherapy during pregnancy	
Yes	43(43%)
No	57(57%)
Beginning time of chemotherapy	
1st	0
2nd	29(67.4%)
3rd	3(7.0%)
Missing	11(25.6%)
Type of chemotherapy	43
Single-agent	7(16.3%)
Double-agent combined	19(44.2%)
Three-drug combined	16(37.2%)
Missing	1(2.3%)
Staging surgery after pregnancy	49
Fertility-sparing surgery	11(22.4%)
Radical surgery	34(69.4%)
Missing	4(8.2%)

Notes: Numbers with percentages are shown.

## Data Availability

All data are either public or available for research upon request without permission.
